# Application of Pyrosequencing Method for Investigating the Diversity of *Synechococcus* Subcluster 5.1 in Open Ocean

**DOI:** 10.1264/jsme2.ME13063

**Published:** 2013-12-28

**Authors:** Dong Han Choi, Jae Hoon noH, Jung-Hyun Lee

**Affiliations:** 1 Marine Biotechnology Research Division, Korea Institute of Ocean Science and Technology, Ansan 426–744, Republic of Korea; 2 Marine Ecosystem Research Division, Korea Institute of Ocean Science and Technology, Ansan 426–744, Republic of Korea

**Keywords:** diversity, internal transcribed spacer (ITS), phylogeny, pyrosequencing, *Synechococcus*

## Abstract

*Synechococcus* are distributed throughout the world’s oceans and are composed of diverse genetic lineages. However, as they are much less abundant than *Prochlorococcus* in oligotrophic open oceans, their in-depth genetic diversity cannot be investigated using commonly used primers targeting both *Prochlorococcus* and *Synechococcus*. Thus, in this study, we designed a primer specific to the 16S–23S rRNA internal transcribed spacer (ITS) of the *Synechococcus* subcluster 5.1. Using the primer, we could selectively amplify *Synechococcus* sequences in oligotrophic seawater samples. Further, we showed that a barcoded amplicon pyrosequencing method could be applicable to investigate *Synechococcus* diversity using sequences retrieved in GenBank and obtained from environmental samples. Allowing sequence analyses of a large number of samples, this high-throughput method would be useful to study global biodiversity and biogeographic patterns of *Synechococcus* in marine environments.

Together with *Prochlorococcus*, *Synechococcus* are the dominant picocyanobacteria in marine environments and are important primary producers (17, 24). *Synechococcus* is distributed ubiquitously throughout the world’s oceans, from equatorial to subpolar waters and from coastal to open ocean areas (36). While *Synechococcus* is most dominant picocyanobacteria in mesotrophic coastal waters, its abundance is far lower than that of *Prochlorococcus* in most oligotrophic open oceans.

For the genetic diversity of picocyanobacteria, 16S–23S internal transcribed spacer (ITS) sequences have been widely used (1, 5, 6, 14, 26, 27, 29). Until now, at least 20 clades belonging to the *Synechococcus* subcluster 5.1 (SC5.1), 2 clades in the *Synechococcus* subcluster 5.2 (SC5.2) and 6 clades in the *Synechococcus* subcluster 5.3 (SC5.3) have been identified from marine environments. Among them, *Synechococcus* SC5.1 has been found to be ubiquitous and to contain dominant genotypes in marine environments (14, 27, 35, 36). However, *Synechococcus* diversity in oligotrophic open oceans, especially in *Prochlorococcus*-dominating environments, has not yet been resolved in detail due to methodological limitations. Among sequences obtained by the commonly used primers targeting marine picocyanobacteria (29), the clone number of *Synechococcus* sequences is too small to resolve their detailed diversity in oligotrophic oceans. In addition, dot-blot hybridization (9, 10, 35) and quantitative PCR using clade-specific primer/probe sequences (2), could not reveal the diversity of clades without available specific primer/probes. Knowledge about the distribution of each *Synechococcus* clade is essential to understand the physiological and genetic adaptations of diverse lineages to a specific niche and their niche partitioning in global oceans (1, 2, 30, 32). In addition, their distribution in open oceans is important to understand the temporal and spatial dynamics of picocyanobacterial diversity by advection, adaptation and competition in boundary waters of marginal seas affected by oligotrophic open oceans (8). Recently, high-throughput pyrosequencing of PCR amplicons has been widely used to reveal the microbial diversity found in various marine environments. This approach allows many samples to be analyzed at a time and produces a large number of sequences for community analyses.

Thus, in this study, we showed that the genetic diversity of *Synechococcus* SC5.1 could be revealed in oligotrophic oceans by a barcoded amplicon pyrosequencing method using a newly designed *Synechococcus* SC5.1-specific primer. The *Synechococcus* lineage distribution in oligotrophic oceans will improve knowledge of niche partitioning, transport and adaptation across water masses in marginal seas, and the response to environmental changes of diverse *Synechococcus* clades.

## Materials and Methods

### Phylogenetic analysis and database construction

To perform phylogenetic analyses of 16S–23S ITS sequences, 16S–23S ITS sequences from marine *Synechococcus* and *Prochlorococcus* were collected from 14 previous studies (1, 3, 5, 6, 12–14, 18–20, 23, 26, 27, 29) and unpublished data (GenBank accession No. HQ336805–HQ336939; Choi & Noh, unpublished data). A sequence from *Synechococcus* KORDI-100 (accession number, KC192550), isolated from the surface of the tropical Pacific Ocean (9°15.95′ N, 158°24.10′ E) in September, 2007 was included in the analysis. A total of 2,024 ITS sequences were aligned using the MAFFT program (version 6) with the FFT-NS-I strategy (21). After manual corrections, the alignment was imported into the ARB program (25). Among environmental sequences, putative chimeric sequences were analyzed using the UCHIME program implemented in the Mothur program (31) where an alignment with only sequences from isolates was used as a reference. After removing chimera, the alignment was manually corrected and a neighbor-joining tree with bootstrap support was constructed using a Mega 5 program (34). Using the tree, the sequences belonging to *Synechococcus* SC5.1 were classified according to previous clade designations (1, 5, 6, 14, 27, 29) and sequences not robustly clustered into any known clades or solely formed an outer branch were removed. Through these procedures, 792 sequences that clustered into *Synechococcus* SC5.1 were finally selected. Phylogenetic trees for the finally selected ITS sequences were constructed using the Mega 5 program for the neighbor-joining method and using the PhyML program (version 3.0) (11) for the maximum-likelihood method. The robustness of tree topologies was assessed by bootstrap analyses based on 100 replications for the neighbor-joining tree and a LRT for maximum-likelihood tree, respectively.

### Sample collection and DNA extraction

Water samples were collected from three stations (Stns) in the northwestern Pacific in May, 2010 aboard the R/V Onnuri. Stn P3 (18°17.4′ N, 134°11.6′ E) is located in an oligotrophic tropical area affected by the oligotrophic North Equatorial Current (NEC). Stns A1 (34°10.0′ N, 128°27.1′ E) and A5 (32°19.6′ N, 126°50.5′ E) are located in the East China Sea, where coastal currents and a branch current of the Kuroshio Current meet, forming oceanic fronts in the central continental shelf region (16). At each station, seawater was sampled using Niskin bottles attached to a rosette sampler. To collect microorganisms, 2 L samples of water were passed through a 0.2 μm Supor filter (Gelman Sciences, Port Washington, NY, USA) and the filters were frozen at −80°C after adding 1 mL STE buffer (100 mM NaCl, 10 mM Tris HCl, 1 mM EDTA, pH 8.0). In the laboratory, the filters were thawed, and DNA was extracted using lysozyme solution, sodium dodecyl sulfate, and proteinase K (33), and then purified using the DNeasy Blood and Tissue Kit (Qiagen, Valencia, CA, USA), following the manufacturer’s instruction. The water temperature and picocyanobacterial abundances in each sample are shown in Table 1.

### Primers and pyrosequencing method

To amplify the partial 16S–23S ITS sequences belonging to *Synechococcus* SC5.1, a reverse primer specific to *Synechococcus* SC5.1 was designed (Table 2). The primer showed a perfect match to 97% of sequences belonging to SC5.1 and mismatches over four bases to *Synechococcus* SC5.2 and SC5.3, and *Prochlorococus* (data not shown). When the reverse primer was used with a previous forward primer targeting picocyanobacterial sequences (ITS-af, Table 2), the amplicon size of the PCR product was expected to be ca. 350 bp. Despite a relatively short amplicon size, this primer set was selected for pyrosequencing application.

Barcoded primers for amplicon pyrosequencing of ITS sequences are shown in Table 2. A total of 1–10 ng template DNA was added to the PCR reaction (total of 50 μL), which contained Ex Taq buffer, 0.2 mM each deoxyribonucleoside triphosphate, 0.5 μM each primer, and 2 units Ex Taq (Takara, Otsu, Japan). PCR amplification was conducted according to the following cycle parameters: an initial denaturation step (5 min, 94°C) was followed by 35 cycles consisting of a denaturation step (45 s, 94°C), annealing (45 s, 50°C), and an extension step (1.5 min, 72°C), with a final 10 min extension step at 72°C at the end. When PCR produced a faint band, nested PCR was applied. In the case, 1st PCR was conducted using the 16S-1247F and 23S-241R primer set (29). The size of the PCR products was confirmed by agarose gel electrophoresis. Each PCR product was quantified on agarose gels using DNA QuantLadders (Lonza Rockland, Rockland, ME, USA). After pooling the same quantities of each PCR product, the pooled PCR product was purified using an AccuPrep PCR purification kit (Bioneer, Daejeon, Korea). After the purified DNA was resolved on a 2% agarose gel, the gel between 350 and 450 bp was excised and DNA was extracted using a Qiagen gel extraction kit (Qiagen). The PCR products were pyrosequenced on a GS-FLX Titanium system (454 Life Sciences, Branford, CT, USA) at Macrogen (Seoul, Korea). Pyrosequencing was run on a 1/8 PicoTiterPlate. In the run, 8 samples used in this study were analyzed together with 66 samples from another study. From the run, 52,273 reads were obtained and were deposited in the NCBI sequence read archive (SRA, http://www.ncbi.nlm.nih.gov/Traces/sra; accession number SRA052533).

### Database and simulation analysis of pyrosequencing data

Pyrosequencing data were mainly analyzed using Mothur software (31). The program can screen bad-quality reads, de-noise pyrosequencing errors, partition reads into each sample based on a barcode sequence, and find chimera using chimera-searching algorithms. Furthermore, provided with reference alignment and taxonomy files, reads can be aligned and assigned to each clade. Thus, from the above ARB database, we constructed another database containing sequences (1,651 sequences) belonging to three *Synechococcus* subclusters and *Prochlorococcus*. Then we annotated the sequences with 4-rank taxonomic information (Cyanobacteria; *Synechococcus* or *Prochlorococcus; Synechococcus* subclusters or *Prochlorococcus* HL/LL; clades). For the reference alignment, the region between the forward primer and tRNA^Ile^ was selected. Because the highly conserved tRNA region was not informative in phylogenetic analysis, the tRNA sequence was removed. In addition, the reference sequence file and the corresponding taxonomic file to be used for clade assignment of sequences were exported from the database.

Simulation analysis was performed to determine whether short ITS sequences obtained by pyrosequencing (ca. 180 bp after removing forward primer and tRNA^Ile^ region) could be correctly classified by analysis with the Mothur pipeline. The *Synechococcus* SC5.1 sequences, in which the clade was determined from phylogenetic analyses using full-length ITS sequences, were randomly divided into two groups using the PASW Statistics 18 program (SPSS, Chicago, IL, USA): one group for the simulation dataset and the other for a test-reference database. The simulation dataset trimmed to both primer regions and the test-reference database was used to make a reference alignment file and a taxonomy file for the simulation analysis. The simulation dataset was aligned using this reference alignment, and clades were assigned by the *k*-nearest neighbor method with an option of *k* = 1 using the ‘classify.seqs’ command in the Mothur program.

In addition, during a pre-clustering step (‘pre.cluster’ command), reads were clustered based on the option ‘diff = 4’ in field data analysis (see below). Applying this option to the simulation dataset, all clusters included sequences from the same clades (data not shown). Thus, sequences from different clades would not be clustered together by pre-clustering.

### Pyrosequencing data analysis of field samples

To analyze the data from field samples, raw reads were filtered to remove reads associated with errors by allowing only a perfect match to the barcode and forward primer sequences. The allowed number of maximum homopolymers was 6. Reads with initial noisy flow (0.5–0.7) before 150 were removed and flows beyond 350 were ignored. Then, flowgram data were grouped by samples based on their barcodes. The filtered reads were de-noised using the ‘shhh. flows’ command, which is the Mothur-based re-implementation of PyroNoise (28, 31). Then the ‘chimera.perseus’ command was used to identify chimeric sequences. The remaining reads were aligned with the Needleman algorithm using the reference alignment. In this step, reads showing similarities less than 90% to reference sequences and short reads not covering full alignment ranges were removed to avoid a possible inconsistent classification by size difference. Using the ‘pre.cluster’ command (with the option of diffs = 4), the aligned reads were clustered to remove sequences that were likely due to pyrosequencing errors. At this stage, chimeric sequences were removed again using the ‘chimera.uchime’ command. The remaining reads were classified to each corresponding clade by the ‘classify.seqs’ command (*k*-nearest neighbor approach with an option of *k* = 1) using the reference sequence and its taxonomic files.

### Other analyses

Picocyanobacterial abundances were determined using a Beckman-Coulter Altra flow cytometer (4). Seawater temperature was measured using a CTD (SBE911; Sea-Bird, Bellevue, WA, USA) mounted on a rosette sampler.

## Results and Discussion

### Phylogenetic analysis of *Synechococcus* SC5.1

**Fig. 1:**
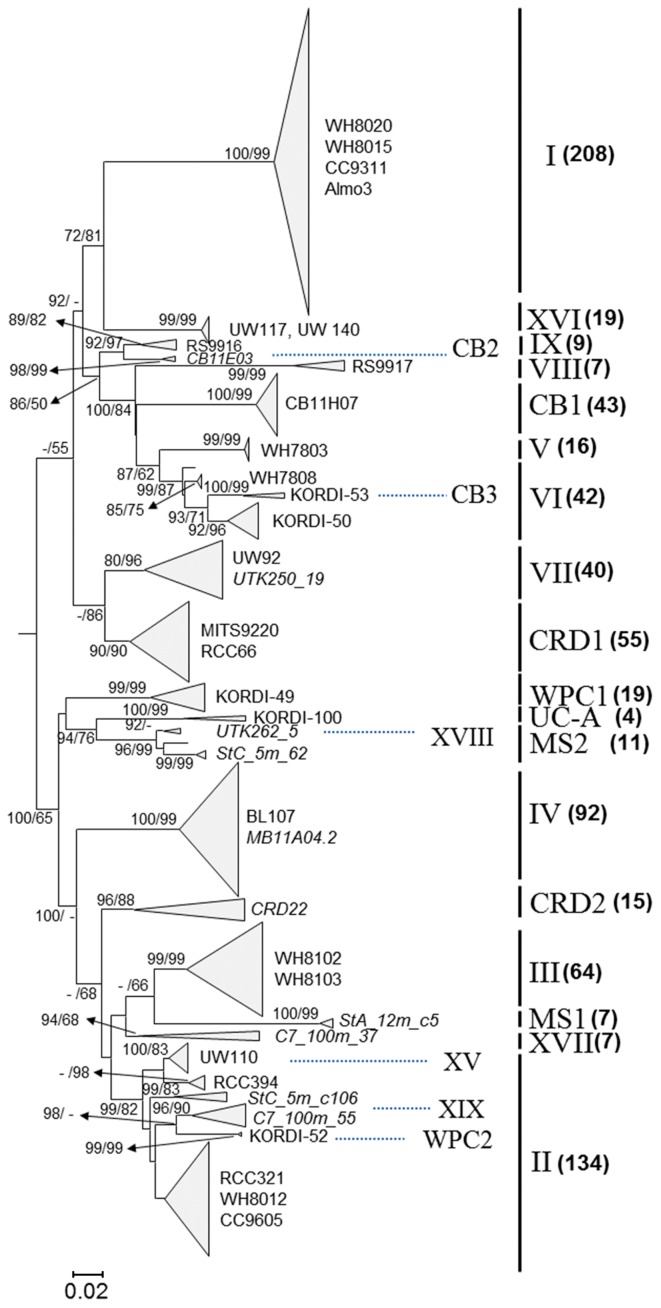
Neighbor-joining (NJ) tree based on 899 positions of the 16S–23S rDNA ITS sequences. A maximum-likelihood (ML) tree was also constructed using the PhyML program (ver. 3.0) with the HKY+i+g model (not shown). Numbers at nodes represent bootstrap values where bootstrap support is >60% in at least one method. Clade names are shown on the right of the vertical lines. Numbers in parentheses represent the number of sequences belonging to each clade. Previously designated clades CB2, CB3, XVIII, XV, XIX and WPC2 were grouped together with other clades (see text). In these cases, the earliest published clade names were selected. Four sequences belonging to *Synechococcus* 5.3 were used as an outgroup (not shown).

In a tree constructed using the full-length 16S–23S ITS sequences, 18 robust clades were identified (Fig. 1). Many previously designated clades formed distinct branches with strong bootstrap support and had relatively long distances among clades (Table S1). However, the inter-clade mean distances between CB2 and IX, between CB3 and VI, and between XVIII and MS2 were relatively low (0.05–0.07) compared to the distances (>0.1) between other robust clades (Table S1). In addition, clades XV, XIX, WPC2, and II formed a branch with high bootstrap support (Fig. 1). Furthermore, the inter-clade mean distances among them were also relatively low, ranging from 0.07 to 0.10, suggesting that they could be treated as one robust clade. In most phylogenetic analyses performed in previous studies (5, 6, 14), not all available ITS sequences were included and evolutionary distances among clades were not considered. This resulted in synonymous clade names.

In this analysis, strain KORDI-100, isolated from the tropical Pacific Ocean, was included and formed a robust clade (UC-A) with two clone sequences from the South China Sea (accession no. HQ849970 and HQ724209; 14, 20) (Fig. 1). Clade UC-A appeared to be a sister clade of MS2, but the inter-clade distance between them was high (0.16; Table S1). The UC-A clade occupied a significant fraction of the *Synechococcus* community in the tropical NW Pacific (see below).

### Methodological consideration of pyrosequencing

In the high-throughput pyrosequencing analyses of microbial diversity, hypervariable regions of the 16S rRNA gene have been used to assess microbial community diversity and have provided equivalent taxonomies and measures of the relative abundance of microbial communities compared to full-length rRNA sequences (15). Likewise, to apply the pyrosequencing method to the study of picocyanobacterial diversity, short amplicons produced by pyrosequencing primers should give sufficient information to assign each read to clades. Although sequence distances between clades slightly decreased in the pyrosequencing region compared to the full-ITS region, mean between-clade distances were still >0.1 and much higher than mean within-clade distances in most cases (Tables S1 and S2).

Furthermore, in the simulation test, the clade classification using the pyrosequencing region was identical to the classification using the full-ITS region (Table 3), suggesting that the 16S–23S ITS sequence fragment could provide sufficient information for discriminating *Synechococcus* clades.

### Application of pyrosequencing to field samples

In the analyses of eight amplicon libraries, 6,003 reads passed our trimming and filtering procedures. Among these, 65 reads were found to be putative chimeras and thus were removed. Additionally, 103 reads showing low sequence similarities less than 90% to reference sequences and 49 short reads having gaps at start or end of alignment were removed. The reads with low sequence similarities to reference sequences might be real members of the known clades. Among them, however, the number of reads targeting *Synechococcus* as the closest relative was only 40 reads and their similarities to reference sequences were low (mean ± SD of 84.7 ± 5.5; data not shown). Further, in the simulation test, the similarities to each closest reference sequence were higher than 90% (Table 3). Thus, missing classification due to the similarity criterion would be negligible.

A total of 5,786 reads passed the screening procedures and 96% of the reads could be assigned to one of the *Synechococcus* clades using the *k*-nearest neighbor algorithm (Table 4). Despite the extremely high ratio of *Prochlorococcus* to *Synechococcus* abundance of ca. 10,000:1 at a depth of 108 m at Stn P3 (Table 1), 76% of the reads belonged to *Synechococcus* SC5.1 (Table 4), suggesting that the primers used in this study could biasedly amplify sequences of *Synechococcus* SC5.1.

However, the possibility of misclassification could not be excluded in some sequence reads with relatively low similarities to reference sequences. To examine this, phylogenetic analysis was additionally conducted. When the 248 representative reads after pre-clustering were added to the existing reference tree using the ARB program (Fig. S1), most of the reads, except 4 representative reads, (total of 5 reads) belonged to the same clade as when classified by the *k*-nearest neighbor algorithm. In company with the above simulation test, these results indicate that *Synechococcus* diversity could be precisely elucidated at the level of clades through pyrosequencing and subsequent data analyses.

By the pyrosequencing approach, we could obtain a large number of *Synechococcus* sequences from the *Prochlorococcus*-dominant oligotrophic NW Pacific Ocean (Table 4). In a previous picocyanobacterial diversity study conducted in surface samples from the NEC area, we could obtain only a small number of *Synechococcus* sequences, with less than 30 reads (8). In this study, on the contrary, ca. 1,000 *Synechococcus* sequences were obtained at the surface of Stn P3 located in the oligotrophic NEC area and thus *Synechococcus* diversity could be confidently analyzed.

Further, the composition of *Synechococcus* SC5.1 was largely congruent with previous knowledge. In coastal and open ocean samples, reads belonging to 11 clades were retrieved (Table 4) and the relative clade distribution showed distinct patterns among samples. At Stns A1 and A5, located in the continental shelf area of the East China Sea, both cold-adapted clades I and IV, and warm-adapted clade II were dominant together (35, 36; Table 4). In previous studies, clades I and IV co-occurred and were found to be dominant in cold coastal water (27, 35, 36). In contrast, clade II was abundant in warm water and present in both coastal and open-ocean water (26, 27, 36). Thus, the observed overlap of cold- and warm-adapted clades at these stations might be due to the mixing of cold coastal water and warm open ocean water in the boundary waters (7, 16). Conversely, in oligotrophic tropical oceans, clade II was predominant with a distinct depth distribution pattern (Table 4) but cold-adapted clades I and IV were not found. Consistently, clade II was highly abundant at warmer and offshore stations (14, 26, 35, 36). Furthermore, new information was obtained. The novel clade UC-A occupied a significant fraction (4–7%) of the surface euphotic zone of oligotrophic oceans (Table 4). As sequences belonging to clade UC-A were very rarely found in previous works (14, 30) and culture strains were isolated from the tropical oligotrophic Pacific Ocean, clade UC-A seems to inhabit oligotrophic open oceans with relatively low *Synechococcus* abundance. In addition, distinct depth distribution among *Synechococcus* clades could be found. The relative percentages of clade II tended to decrease with depth, whereas clades III, XVII, and CRD1 appeared at depth (Table 4). This depth-partitioning pattern might be related with the differential responses of each clade to environmental conditions such as light availability and nutrients, similar to *Prochlorococcus* distributional patterns (29, 30, 35), and competition among clades. However, more data will be needed to elucidate the depth partitioning of *Synechococcus* clades in oligotrophic oceans.

Therefore, these results showed that in-depth *Synechococcus* lineage diversity in open oceans as well as in coastal waters could be revealed using a *Synechococcus*-specific primer and the pyrosequencing approach. Dot-blot hybridization and qPCR have been applied to reveal picocyanobacterial diversity in marine environments (2, 35, 36) and thus might be applied to oligotrophic oceans. However, more clade-specific probe/primer sequences should be developed to fully resolve their diversity. Further, even if clade-specific probes for all known clades are developed, the application of dozens of probes to elucidate the whole diversity would be time-consuming as well as expensive. In contrast, the pyrosequencing method can obtain tens of thousands of sequences from a single run of over 100 amplicon libraries on a 1/8 PicoTiter Plate. Thus, as seen in this study, pyrosequencing is one of the preferred tools for studying in-depth picocyanobacterial diversity in a large number of samples as it is a labor- and time-saving, and thus cost-efficient method as well as providing a higher quantity of sequencing reads.

In conclusion, to understand the temporal and spatial distribution, global biogeography and ecological niches of *Synechococcus* lineages, it is necessary to accumulate knowledge on *Synechococcus* diversity in a variety of environments. The barcoded amplicon pyrosequencing method developed in this study will be useful to elucidate the fine-scale biogeographical distribution of *Synechococcus* diversity in marine environments.

## Supplementary material



## Figures and Tables

**Table 1:** Water temperature and picocyanobacterial abundance measured in each sample

Station	Sampling depth (m)	Temperature (°C)	*Synechococcus* (×10^3^ cells ml^−1^)	*Prochlorococcus* (×10^4^ cells ml^−1^)
A1	0	20.3	94.3	0.01
A5	30	17.6	52.3	Not detected
P3	0	29.5	1.2	2.2
	5	29.5	0.9	2.5
	15	29.5	1.0	2.6
	30	28.6	0.7	2.3
	75	26.8	0.8	8.2
	108	25.5	0.01	13.4

**Table 2:** Primers (adapter+key+barcode+specific oligonucleotides) used to amplify 16S–23S rRNA ITS sequences of *Synechococcus* SC5.1

Primer	Adapter	Key	Barcode	Specific oligonucleotides	Reference for sp. primer
ITS-af-fusion (forward)	CCATCTCATCCCTGCGTGTCTCCGAC	TCAG	Variable (10-mers§)	GGATCACCTCCTAACAGGGAG	Lavin *et al.* (22)
Syn-ar-fusion* (reverse)	CCTATCCCCTGTGTGCCTTGGCAGTC	TCAG	none	AGGTTAGGAGACTCGAACTC	This study

* Specific oligonucleotide sequence of this primer is specific to *Synechococcus* subcluster 5.1 and located on tRNA^Ala^ gene (*see* text for more details).

§ Barcode sequences supplied by Roche were used.

**Table 3:** Clade assignment of a simulation dataset using the Mothur pipeline

Clade	No. of sequences		Similarity to reference sequence (ranges)

	Analyzed using Full-ITS region*	Analyzed using pyrosequencing region	
I	114	114	97.2–100
II	59	59	97.7–100
III	21	21	97.8–100
IV	48	48	96.6–100
V	10	10	98.3–100
VI	22	22	97.2–100
VII	19	19	97.2–100
VIII	3	3	99.4–100
IX	4	4	98.3–100
XVI	8	8	98.9–100
XVII	4	4	94.4–99.4
CB1	26	26	97.8–100
CRD1	26	26	93.8–100
CRD2	5	5	96.6–100
MS1	3	3	98.9–100
MS2	5	5	96.6–100
WPC1	9	9	96.7–100
UC-A	2	2	94.9–97.7

* The phylogenetic positions of each sequence were defined using full-ITS sequences.

**Table 4:** Numbers and percentages of reads belonging to each clade obtained in this study

Clade		Reads No./%	Station/Depth (m)							

			A1	A5	P3					
	
			0	30	0	5	15*	30	75	108*
*Synechococcus* SC-5.1	I	155	124	31	0	0	0	0	0	0
		%	**26.0**	**13.9**	**0**	**0**	**0**	**0**	**0**	**0**
	II	4250	59	103	1064	884	1286	388	230	236
		%	**12.4**	**46.2**	**90.5**	**88.8**	**89.4**	**80.2**	**75.7**	**50.4**
	III	95	0	0	1	9	3	22	43	17
		%	**0**	**0**	**0.1**	**0.9**	**0.2**	**4.5**	**14.1**	**3.6**
	IV	381	294	87	0	0	0	0	0	0
		%	**61.6**	**39.0**	**0**	**0**	**0**	**0**	**0**	**0**
	VII	15	0	1	0	0	0	0	1	13
		%	**0**	**0.4**	**0**	**0**	**0**	**0**	**0.3**	**2.8**
	XVI	1	0	1	0	0	0	0	0	0
		%	**0**	**0.4**	**0**	**0**	**0**	**0**	**0**	**0**
	XVII	171	0	0	2	0	9	0	10	150
		%	**0**	**0**	**0.2**	**0**	**0.6**	**0**	**3.3**	**32.1**
	CRD1	56	0	0	10	6	20	14	0	6
		%	**0**	**0**	**0.9**	**0.6**	**1.4**	**2.9**	**0**	**1.3**
	CRD2	28	0	0	11	0	0	10	6	1
		%	**0**	**0**	**0.9**	**0**	**0**	**2.1**	**2.0**	**0.2**
	WPC1	220	0	0	43	49	62	15	6	45
		%	**0**	**0**	**3.7**	**4.9**	**4.3**	**3.1**	**2.0**	**9.6**
	UC-A	194	0	0	45	48	58	35	8	0
		%	**0**	**0**	**3.8**	**4.8**	**4.0**	**7.2**	**2.6**	**0.0**
	Sum	5566	477	223	1176	996	1438	484	304	468
*Prochlorococcus*		220	0	0	0	1	67	0	0	152

* Samples amplified by nested PCR.
