# Supplementary material for: Application of Pyrosequencing Method for Investigating the Diversity of Synechococcus Subcluster 5.1 in Open Ocean
Source: Microbes Environ. 2013 Dec 28;29(1):17–22. doi: 10.1264/jsme2.ME13063 (PMC4041225; doi:10.1264/jsme2.ME13063)

Supplementary Table S1. Mean within-clade (bold text in diagonal cells) and between-clade p-distances. Distances were calculated for 899 positions of 16S-23S rRNA ITS sequences except two t-RNA sequences using the Mega 5 program. Phylogenetic relationships among each clade are shown in Figure 1.

	II	XV	WPC2	XIX	VI	CB3	IX	CB2	MS2	XVIII	I	III	IV	V	VII	VIII	XVI	CRD1	CRD2	CB1	WPC1	XVII	MS1	UC-A
**II**	**0.046**																							
**XV**	0.071	**0.028**																						
**WPC2**	0.072	0.087	**0.005**																					
**XIX**	0.099	0.089	0.088	**0.046**																				
**VI**	0.192	0.195	0.198	0.184	**0.031**																			
**CB3**	0.178	0.195	0.196	0.194	0.074	**0.021**																		
**IX**	0.165	0.158	0.183	0.185	0.147	0.159	**0.027**																	
**CB2**	0.173	0.166	0.201	0.197	0.145	0.159	0.062	**0.016**																
**MS2**	0.152	0.118	0.164	0.158	0.217	0.222	0.172	0.181	**0.012**															
**XVIII**	0.144	0.109	0.159	0.152	0.203	0.208	0.167	0.176	0.053	**0.025**														
**I**	0.247	0.237	0.262	0.259	0.217	0.227	0.182	0.184	0.248	0.237	**0.037**													
**III**	0.131	0.139	0.136	0.149	0.218	0.228	0.200	0.215	0.199	0.190	0.277	**0.043**												
**IV**	0.177	0.173	0.175	0.160	0.244	0.241	0.243	0.242	0.209	0.207	0.260	0.201	**0.062**											
**V**	0.188	0.198	0.199	0.188	0.091	0.129	0.138	0.138	0.215	0.202	0.222	0.213	0.245	**0.001**										
**VII**	0.196	0.175	0.187	0.194	0.176	0.174	0.129	0.142	0.187	0.181	0.218	0.208	0.213	0.187	**0.061**									
**VIII**	0.232	0.243	0.238	0.247	0.186	0.219	0.176	0.181	0.246	0.240	0.263	0.279	0.260	0.174	0.209	**0.038**								
**XVI**	0.202	0.193	0.215	0.201	0.161	0.175	0.124	0.136	0.204	0.191	0.183	0.211	0.220	0.166	0.164	0.224	**0.006**							
**CRD1**	0.205	0.199	0.213	0.210	0.194	0.191	0.141	0.158	0.196	0.187	0.210	0.204	0.221	0.188	0.124	0.224	0.168	**0.062**						
**CRD2**	0.143	0.135	0.160	0.158	0.235	0.231	0.185	0.204	0.189	0.184	0.259	0.162	0.197	0.228	0.181	0.274	0.207	0.184	**0.097**					
**CB1**	0.218	0.230	0.224	0.225	0.145	0.153	0.150	0.149	0.239	0.234	0.238	0.245	0.265	0.140	0.193	0.199	0.190	0.210	0.258	**0.014**				
**WPC1**	0.158	0.168	0.179	0.173	0.228	0.219	0.199	0.212	0.169	0.170	0.249	0.175	0.197	0.229	0.188	0.262	0.187	0.207	0.192	0.242	**0.037**			
**XVII**	0.168	0.163	0.186	0.174	0.247	0.245	0.209	0.218	0.207	0.204	0.270	0.181	0.219	0.236	0.204	0.280	0.214	0.208	0.194	0.260	0.184	**0.114**		
**MS1**	0.162	0.164	0.179	0.179	0.243	0.251	0.238	0.240	0.224	0.218	0.304	0.168	0.231	0.238	0.229	0.296	0.235	0.242	0.198	0.265	0.188	0.183	**0.014**	
**UC-A**	0.205	0.200	0.214	0.213	0.259	0.260	0.222	0.224	0.155	0.150	0.260	0.203	0.222	0.249	0.204	0.273	0.231	0.204	0.209	0.260	0.190	0.206	0.205	**0.062**

Supplementary Table S2. Mean within-clade (bold text in diagonal cells) and between-clade p-distances. Distances were calculated for 222 aligned positions between the forward primer and $tRNA^{Ile}$.

	I	II	III	IV	V	VI	VII	VIII	IX	XVI	XVII	CB1	CRD1	CRD2	MS1	MS2	WPC1	UC-A
I	**0.031**																	
II	0.144	**0.065**																
III	0.187	0.147	**0.030**															
IV	0.212	0.175	0.193	**0.038**														
V	0.153	0.127	0.141	0.208	**0.004**													
VI	0.135	0.122	0.154	0.196	0.058	**0.039**												
VII	0.175	0.111	0.142	0.171	0.160	0.154	**0.078**											
VIII	0.200	0.159	0.221	0.219	0.106	0.122	0.176	**0.026**										
IX	0.131	0.084	0.145	0.198	0.078	0.095	0.130	0.143	**0.030**									
XVI	0.159	0.110	0.132	0.188	0.102	0.108	0.146	0.171	0.102	**0.005**								
XVII	0.178	0.119	0.144	0.175	0.147	0.156	0.130	0.188	0.139	0.144	**0.117**							
CB1	0.160	0.125	0.170	0.201	0.120	0.131	0.146	0.162	0.085	0.131	0.169	**0.012**						
CRD1	0.163	0.121	0.155	0.179	0.155	0.157	0.120	0.201	0.129	0.150	0.125	0.169	**0.061**					
CRD2	0.159	0.097	0.146	0.200	0.130	0.138	0.113	0.180	0.108	0.138	0.126	0.151	0.116	**0.057**				
MS1	0.218	0.157	0.134	0.221	0.140	0.162	0.154	0.220	0.169	0.152	0.141	0.183	0.162	0.154	**0.014**			
MS2	0.149	0.084	0.180	0.181	0.144	0.139	0.121	0.174	0.106	0.135	0.134	0.147	0.120	0.113	0.182	**0.034**		
WPC1	0.190	0.107	0.088	0.194	0.147	0.160	0.131	0.207	0.126	0.108	0.114	0.169	0.129	0.110	0.130	0.142	**0.037**	
UC-A	0.208	0.181	0.175	0.225	0.213	0.211	0.170	0.226	0.199	0.197	0.170	0.179	0.174	0.176	0.150	0.191	0.167	**0.063**

Supplementary Figure S1. Phylogenetic tree of the 248 representative 16S-23S ITS sequences retrieved by the pyrosequencing method. The aligned sequences were added to the existing tree constructed on the basis of full-length ITS sequences using ARB program. Only sequences obtained by the pyrosequencing showed their names. Clade names were shown in bold type.

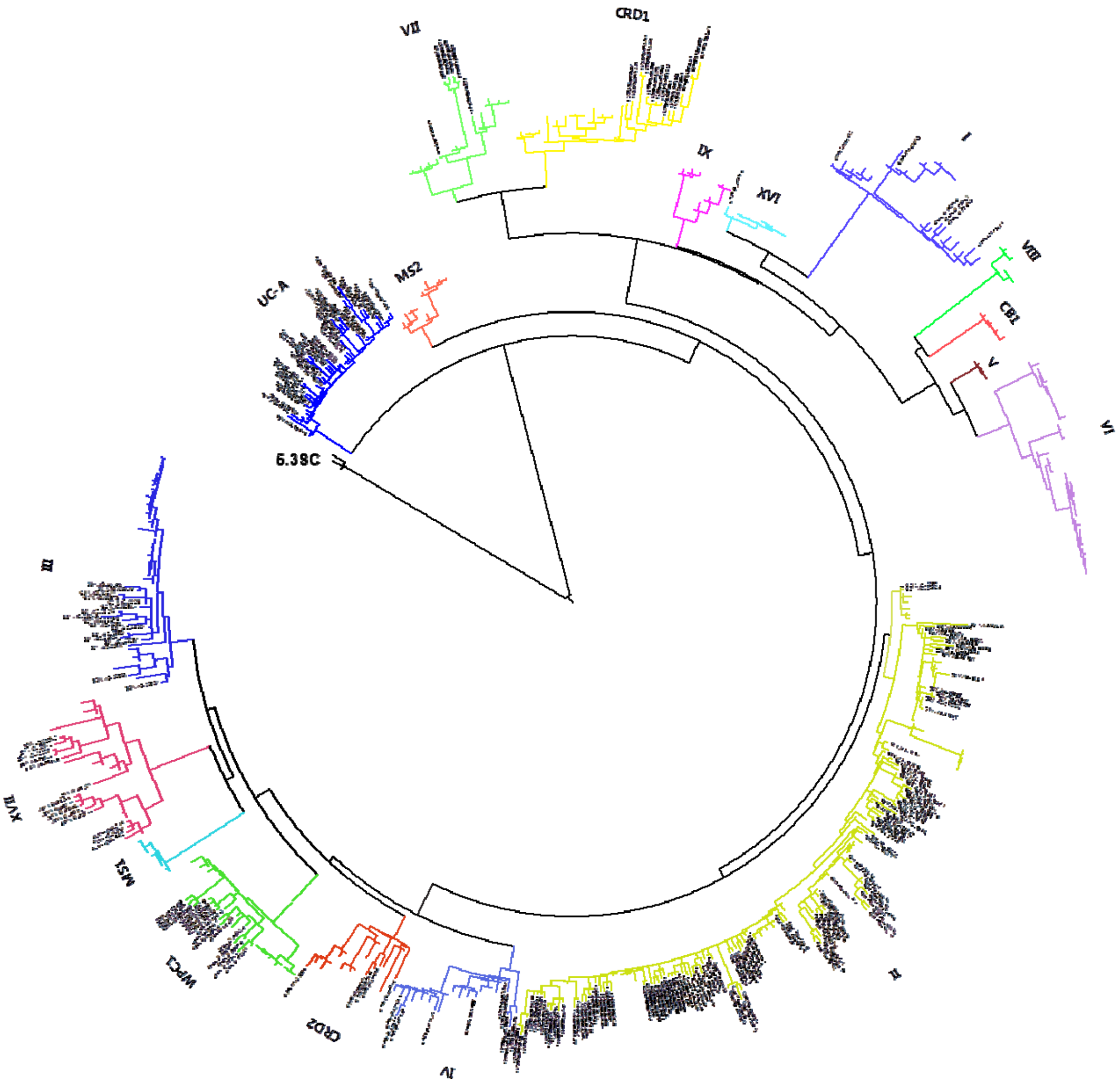

Supplement: Supplementary file 1 [file 29_17_s1.pdf]
